# Saponins From *Paris forrestii* (Takht.) H. Li Display Potent Activity Against Acute Myeloid Leukemia by Suppressing the RNF6/AKT/mTOR Signaling Pathway

**DOI:** 10.3389/fphar.2018.00673

**Published:** 2018-06-26

**Authors:** Qin Lu, Yuanming He, Yuehu Wang, Li Gao, Yunjing Zheng, Zubin Zhang, Biyin Cao, Qi Wang, Xinliang Mao, Shaoyan Hu

**Affiliations:** ^1^Department of Hematology and Oncology, Children’s Hospital of Soochow University, Suzhou, China; ^2^Jiangsu Key Laboratory of Neuropsychiatric Diseases and Department of Pharmacology, College of Pharmaceutical Sciences, Soochow University, Suzhou, China; ^3^Key Laboratory of Economic Plants and Biotechnology, and Yunnan Key Laboratory for Wild Plant Resources, Kunming Institute of Botany, Chinese Academy of Sciences, Kunming, China; ^4^Institute of Clinical Pharmacology, Guangzhou University of Chinese Medicine, Guangzhou, China

**Keywords:** TSPf, RNF6/AKT/mTOR signaling pathway, acute myeloid leukemia, herbal medicine, saponins, cell apoptosis, RNF6

## Abstract

Saponins are amphipathic glycosides found in traditional Chinese medicines. In the present study, we isolated a panel of saponins from *Paris forrestii* (Takht.) H. Li, a unique plant found in Tibet and Yunnan provinces, China. By examining their activities in suppressing acute myeloid leukemia (AML) cell proliferation, total saponins from *Paris forrestii* (TSPf) displayed more potent activity than individual ones. TSPf induced more than 40% AML cell apoptosis and decreased the viability of all leukemia cell lines. TSPf-induced apoptosis was confirmed by both Annexin V staining and caspase-3 activation. In line with these findings, TSPf downregulated pro-survival proteins Mcl-1, Bcl-xL, and Bcl-2 but upregulated the expression of tumor suppressor proteins p53, p27, Bax, and Beclin 1. The AKT/mTOR signaling pathway is frequently overactivated in various AML cells, and TSPf was found to suppress the activation of both AKT and mTOR, but had no effects on their total protein expression. This was further confirmed by the inactivation of 4EBP-1 and p70S6K, two typical downstream signal molecules in the AKT/mTOR pathway. Moreover, TSPf-inactivated AKT/mTOR signaling was found to be associated with downregulated RNF6, a recently identified oncogene in AML. RNF6 activated AKT/mTOR, and consistently, knockdown of RNF6 led to inactivation of the AKT/mTOR pathway. Furthermore, TSPf suppressed the growth of AML xenografts in nude mice models. Oral administration of TSPf almost fully suppressed tumor growth without gross toxicity. Consistent with the findings in cultured cell lines, TSPf also downregulated RNF6 expression along with inactivated AKT/mTOR signaling in tumor tissues. This study thus demonstrated that TSPf displays potent anti-AML activity by suppressing the RNF6/AKT/mTOR pathway. Given its low toxicity, TSPf could be developed for the treatment of AML.

## Introduction

Acute leukemia is a hematological malignancy characterized by uncontrollable and rapid proliferation of malfunctional myeloid or lymphoblastic cells. The past decade has witnessed the advances in the treatment of ALL and currently the survival rate of ALL patients has reached as high as 80–90% (Kato and Manabe, 2017). However, the outcome of AML is still poor. AML is a heterogeneous disease characterized by the accumulation of immature myeloid progenitor cells in the bone marrow, compromising of normal hematopoiesis and ultimately resulting in bone marrow failure (Papaemmanuil et al., 2016). Maine therapies for AML include chemotherapy, radiotherapy, and hematopoietic stem cell transplantation (HSCT). Chemotherapy is the mainstay treatment for all AML patients (Othus et al., 2015), however, drug resistance and clinical relapse limits its efficacy toward AML (Krug et al., 2016). The 5-year survival rate is only 26.6%. Survival rates are even lower among patient ages 65 to 74 years (5.3%) and 75 years or older (1.6%). These elderly patients constitute more than 50% of new AML cases (Sorror et al., 2017). Therefore, exploring novel therapeutic agents is urgent for improving the outcome of patients with AML.

It is well known that the AKT/mTOR axle is a central node for the signal transduction and it is frequently activated in AML thus promoting AML cell proliferation and survival. A plethora of studies have shown that the AKT/mTOR molecules are promising therapeutical targets for cancers (Li et al., 2014; Nitulescu et al., 2016). Currently several AKT inhibitors are under evaluation in clinical trials, while mTOR inhibitors such as temsirolimus and analogs have been approved by US Food and Drug Administration for the treatment of various cancers (Zhu et al., 2014), which light hope for novel ones for AML treatment. The ring finger protein RNF6 is an oncoprotein that was raised recently. It has been found to be highly expressed in several cancers, including prostate cancer (Xu et al., 2009), breast cancer (Zeng et al., 2017), colorectal cancer (Liang et al., 2018; Liu et al., 2018), and AML (Xu et al., 2016). In AML cells, RNF6 promotes AML cell proliferation and tumor growth (Xu et al., 2016). It has been proposed as a therapeutic target for AML therapy (Xu et al., 2016), but therefore are no inhibitors of RNF6 reported.

Natural products are excellent and reliable sources for the development of anti-cancer drugs. Some drugs such as harringtonine from *Cephalotaxus hainanensis* Li have been widely used in AML patients (Zhou et al., 1995; Kantarjian et al., 2015). To search for novel natural products for AML patients, we turned to *Paris forrestii* (Takht.) H. Li, an unique plant in Tibet and Yunnan provinces in China. *Paris forrestii* (Takht.) H. Li has long been used in traditional Chinese medicine and the crude extract from this plant has been used for the treatment of infection, bleeding, even snake biting by local inhabitants. Compared with *Paris forrestii* (Takht.) H. Li, *Paris polyphylla* var. *yunnanensis* has been examined for their anti-tumor activities (Wu et al., 2012; Qin et al., 2016; Jing et al., 2017; Zan et al., 2017). However, the anti-AML activity of *Paris forrestii* (Takht.) H. L has not been reported. In the present study, we isolated and characterized saponins and other major components from this plant and evaluated their anti-AML activities. The results showed that the total saponins markedly induced AML cell apoptosis by inhibiting the RNF6/AKT/mTOR signaling pathway.

## Materials and Methods

### Reagents

Propidium iodide (PI), MTT, RPMI-1640 medium, and fetal bovine serum (FBS) were purchased from Sigma (St. Louis, MO, United States). Annexin V-FITC Apoptosis Detection Kit was purchased from Beyotime Institute of Biotechnology (Nantong, Jiangsu, China).

### Isolation and Identification of Total Saponins From *Paris forrestii* (Takht.) H. Li

The protocol to isolate saponins was adapted from a previous study (Wu et al., 2012). Briefly, dry roots (4.4 kg) from *Paris forrestii* (Takht.) H. Li were processed into powder, followed by soak in 95% of ethanol (13 L) for 7 days and subsequent filtration of the whole mixtures. The throughput of the filtration was subjected to suspension in ddH_2_O and further extracted with ethyl acetate. The solution after ethyl acetate extract was further isolated with *n*-butanol (2L × 3) and the resultant fraction (290 g) was subjected to further isolation by high-performance liquid chromatography (HPLC) and structural determination by mass spectrometry and NMR. HPLC was performed on the Agilent 1200 HPLC with a column Zorbax SB-C18 (9.4 mm × 250 mm). Dependent on the specific fraction, the mobile phases were methanol:water (72:28), acetonitrile:water (50:50), or acetonitrile:water (40:60), respectively. The flow rate was 2 mL/min for all the HPLC assays.

### Cell Culture

Human myeloid leukemia cells (K562, HL-60, KG-1, and HT-93) and lymphoblastic leukemia cells (preB-697 and Jurkat) were purchased from American Type Culture Collection (ATCC, Washington, DC, United States) or maintained in the lab. HEK293T cells were kindly provided by Dr. Michael F. Moran, University of Toronto, Canada. All cell lines were cultured in RPMI-1640 medium (Hyclone), supplemented with 10% fetal calf serum, 100 μg/ml penicillin, and 100 U/ml streptomycin at 37°C with 5% CO_2_ in a humidified incubator.

### Cell Transfection

HEK293T cells at the log phase were transfected with a Flag-RNF6 plasmid in pcDNA3.1 vector using polyethyleneimine (PEI, Sigma-Aldrich Co., St. Louis, MO, United States) as the gene carrier. The detailed protocol was described previously (Wang et al., 2017). Short hairpin RNF6 (shRNF6) was obtained from GeneChem Biotech, Inc., Shanghai, China. Lentiviral RNF6 was generated as described previously (Xu et al., 2016).

### Determination of Cell Viability

Cell viability was determined by the MTT assay as described previously (Walter et al., 2017). AML cells were seeded in 96-well microtiter plates at a density of 10^5^ cells/well. After exposure to various concentrations of saponins or DMSO, 10 μl of MTT (5 mg/ml) was added to each well and cells were further incubated at 37°C for 4 h. DMSO was then added to dissolve formazan crystals, and the absorbance (OD_570_) was determined using a microplate reader (Molecular Device^®^). The IC_50_ values were calculated using GraphPad^®^ Prism 5.

### Apoptosis Analysis by Annexin V-FITC and PI Staining

K562, HL-60, KG-1, and HT-93 cells were treated with TSPf for 24 h. Cells were harvested, washed twice with ice-cold PBS, resuspended with 200 μl of binding buffer containing 5 μl Annexin V-FITC, and incubated in dark for 10 min according to the instructions of the Kit (Beyotime, Shanghai, China). After incubation, the cells were centrifuged at 1000 × *g* for 5 min, resuspended with 200 μl of binding buffer containing 10 μl PI, and then analyzed on a flow cytometry (Beckman Coulter, Epics XL, United States).

### Immunoblotting

Total proteins were extracted from TSPf-treated cells using a 0.5% SDS-containing protein lysis buffer (KeyGEN Biotech, Beijing, China). Protein concentrations were determined by the BCA assay (Beyotime). Forty micrograms proteins from each sample was electrophoresed on 8–12% SDS-polyacrylamide gels and transferred to polyvinylidene fluoride membranes. The resultant blots were incubated at 4°C overnight with the appropriate primary antibody after pre-blocking incubation with 5% non-fat milk. The blots were then probed with an appropriate secondary antibody (1:5000) for 2 h. The following assay was performed as described previously (Wang et al., 2017). Monoclonal antibodies to human PARP, Caspase-3, cleaved Caspase-3, Mcl-1, Bax, Bcl-2, Bcl-xL, p27, p53, Beclin1, RNF6, AKT, p-AKT, mTOR, p-mTOR, P70S6K, p-P70S6K, 4E-BP-1, p-4E-BP-1, p62, and LC3 were purchased from Cell Signaling Technology (Danvers, MA, United States). Antibody against GAPDH and all secondary antibodies were purchased from Santa Cruz Biotechnology (Santa Cruz, CA, United States).

### Gene Expression Data Mining

The association of RNF6 expression with the overall survival of AML patients was analyzed using the PROG gene database^1^ (Goswami and Nakshatri, 2013) based on the AML data from the Cancer Genome Atlas (TCGA)^2^.

### Reverse Transcription Polymerase Chain Reaction (RT-PCR)

Total RNA was extracted from K562 and HL-60 cells using Trizol (Transgene, Beijing, China) as described previously (Xu et al., 2016). cDNA preparation was accomplished with 2 μg RNA using a Superscript^TM^-III kit (Invitrogen) according to the manufacturer’s instruction and described previously (Wang et al., 2017). PCR amplification primers for RNF6 were 5′-CCCGGAATTCATGAATCAGTCTAGATCGAGATCAG-3′ (Forward) and 5′-AAATATGCGGCCGCTTACCCATTGTTTGCTATGTTAGACCC-3′ (Reverse). The primers for GAPDH were 5′-CAAGGTCATCCATGACAACTTTG-3′ and 5′-GTCCACCACCCTGTTGCTGTAG-3′.

### The Leukemia Xenograft Study

K562 cells were applied to establish a human leukemia xenograft model in nude mice as described previously (Xu et al., 2016). Thirty million K562 cells were injected subcutaneously into the right flanks of nude mice (5- to 6-weeks-old, females, The Slac Laboratory Animal Co., Ltd., Shanghai, China). When tumors were palpable, mice were divided randomly into two groups (*n* = 12), one was orally administered TSPf (vehicle: 1% DMSO in RPMI-1640 medium) (100 mg/kg body weight) and another group received the vehicle only. Tumor sizes and body weight were monitored every other day. At the end of the experiment of the *in vivo s*tudies, whole blood samples were collected from the eyes and were immediately subjected to complete blood analysis and biochemical analyses as described previously (Han et al., 2014). This xenograft study was approved by the Review Board on Experimental Animals of Soochow University, Suzhou, China.

### Statistical Analysis

Statistical significance of the differences observed in drug-treated versus control cells or animals was determined using *Student*’s *t*-test. The minimal level of significance was *P* < 0.05. Except for the *in vivo* xenograft study (that was performed twice), all others were carried out at least three times.

## Results

### Identification of Saponins From *Paris forrestii* (Takht.) H. Li

**FIGURE 1:**
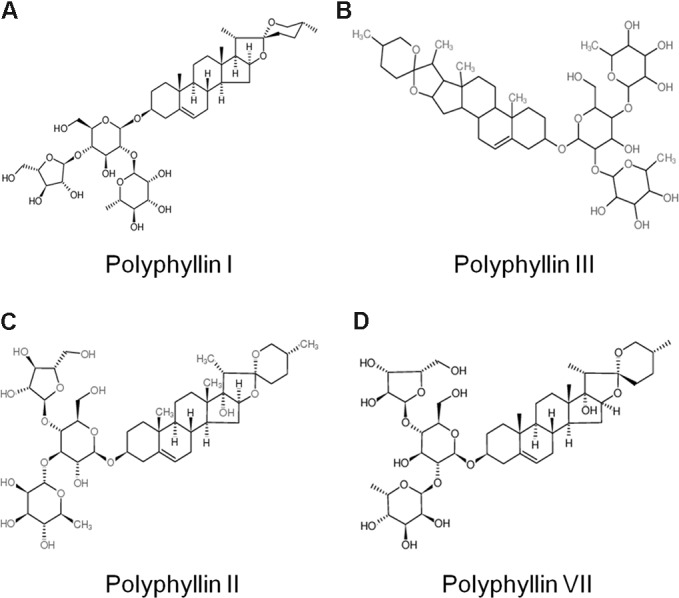
Chemical structure of typical saponins from *Paris forrestii* (Takht.) H. Li. **(A)** Polyphyllin I; **(B)** Polyphyllin II; **(C)** Polyphyllin III; **(D)** Polyphyllin VII.

Saponins are the most abundant and the most active medical ingredients found in *Paris polyphylla* (Zhou, 1991). To identify the active components from *Paris forrestii* (Takht.) H. Li, the dry root of the plant was powdered followed by a sequential extract using solvents ethanol, ethyl acetate, and *n*-butanol. After NMR and MS determination, 10 major compounds (TSPf) were identified from the last *n*-butanol extract, which were polyphyllin I (**Figure 1A**), polyphyllin II (**Figure 1B**), polyphyllin III (**Figure 1C**), polyphyllin V, polyphyllin VII (**Figure 1D**), methyl-prosapogenin I, methyl-prosapogenin V, pariposide A, β-ecdysone, and pennogenin-3-O-α-L-rhamnopyranosyl-(1→2)-[α-L-rhamnopyranosyl-(1→4)-β-D-glucopyranoside. Most of these ingredients have been reported from the roots of *Paris polyphylla* var. *yunnanensis*, except for methyl-prosapogenin I and V (Wu et al., 2012).

### TSPf Is More Active in Suppressing AML Cell Proliferation Than Individual Components

**FIGURE 2:**
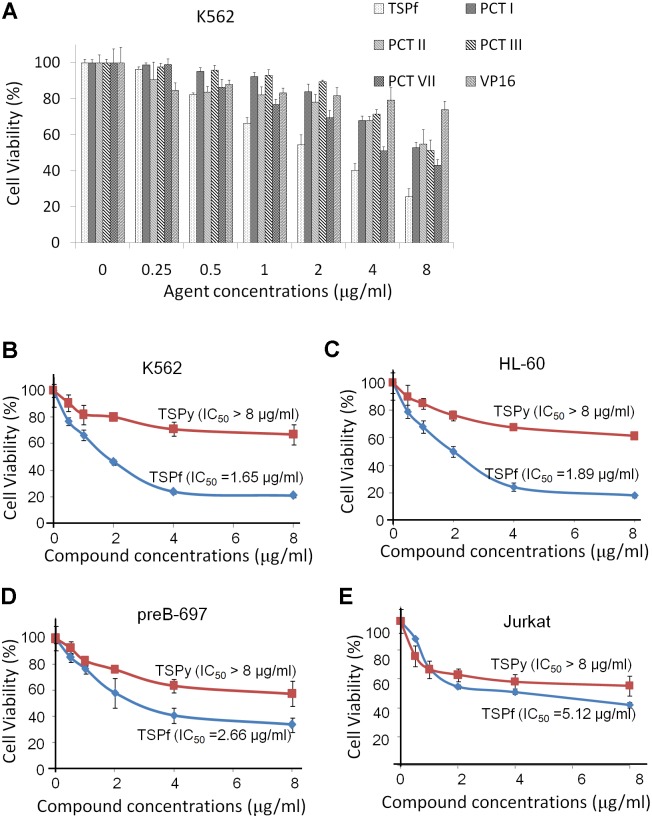
TSPf inhibits the growth of acute myeloid leukemia. **(A)** K562 cells were incubated with representative saponins from *Paris forrestii* (Takht.) H. Li, for 24 h, followed by MTT assay. Human acute leukemia cell lines K562 **(B)**, HL-60 **(C)**, preB-697 **(D)**, and Jurkat **(E)** were treated with TSPf or TSPy at indicated concentrations for 24 h, followed by MTT assay.

As a traditional Chinese medicine, extracts of *Paris forrestii* (Takht.) H. Li have been long been used for the treatment of inflammation, viral infection, and other diseases. To evaluate the effects of individual Paris polyphyllins on AML cell proliferation, total saponins (TSPf), and typical Paris polyphyllins (polyphyllin I, II, III, and VII) were tested in AML cell line K562 using VP-16, a typical anti-AML drug, as the positive control. The results showed that polyphyllin I, II, III, and VII were less potent than TSPf (**Figure 2A**). Interestingly, TSPf displayed strong activity in suppressing proliferation of AML but not in ALL cells. As shown in **Figures 2D,E**, typical lymphoid leukemia cell lines pre-B 697 and Jurkat cells were less sensitive to TSPf. We also compared TSPf with the total saponins from Rhizoma *Paridis* Yunnanensis (TSPy). The results showed that TSPy was less potent than TSPf in suppressing AML cell proliferation (**Figures 2B–D**).

### TSPf Induces Apoptosis of AML Cells

**FIGURE 3:**
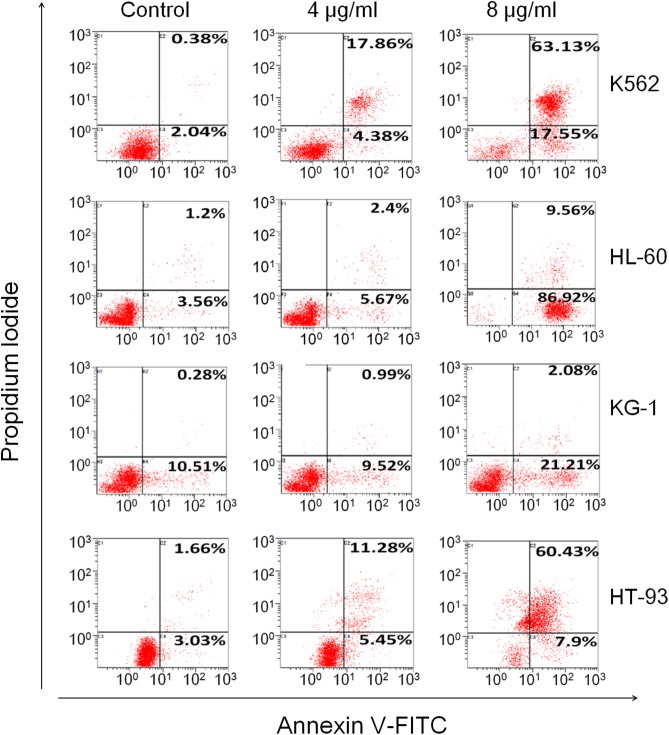
TSPf induces apoptosis of leukemia cells. K562, HL-60, KG-1, and HT-93 cells were treated with TSPf at indicated concentrations for 24 h. Cells were then stained by Annexin V-FITC and propidium iodide and analyzed on a flow cytometer.

To determine whether TSPf also induced AML cell apoptosis, two more AML cell lines KG-1 and HT-93, in addition to K562 and HL-60, were incubated with TSPf from 0 to 8 μg/ml for 24 h before being applied for Annexin V-FITC and PI staining and the analyzed by a flow cytometer. The results showed that TSPf increased Annexin V-positive cells in a concentration-dependent manner (**Figure 3**). In some cell lines such as K562 and HL-60, Annexin V-positive cells accounted for more than 80% (**Figure 3**) after the treatment, suggesting that TSPf induced AML cell apoptosis.

**FIGURE 4:**
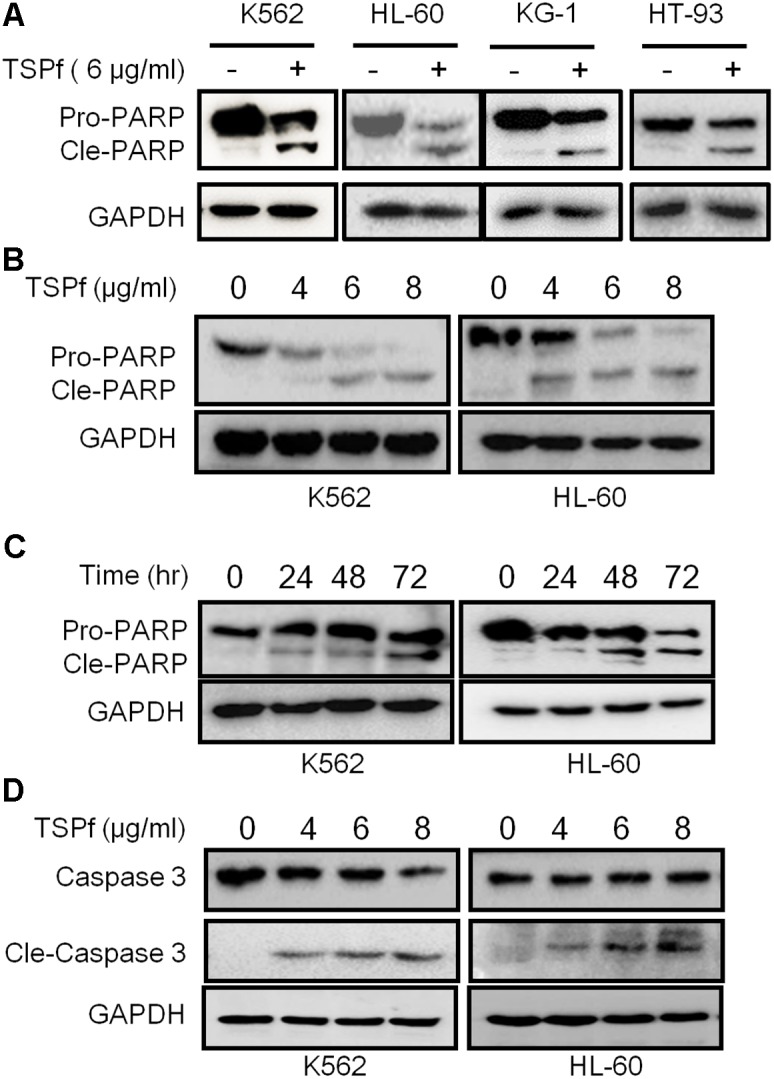
TSPf induces leukemia cell apoptosis. **(A)** Leukemia cell lines were treated with 6 μg/ml TSPf or DMSO for 24 h followed by lysate preparation and immunoblotting analysis against PARP and GAPDH. **(B,C)** K562 and HL-60 cells were treated with TSPf at indicated concentrations **(B)** for 24 h or 4 μg/ml of TSPf for indicated periods **(C)** followed by analysis for PARP. GAPDH was used as a loading control. **(D)** K562 and HL-60 cells were treated with TSPf at indicated concentrations for 24 h followed by immunoblotting assays against caspase 3. GAPDH was used as an internal loading control.

To verify the TSPf-induced apoptosis, K562 and HL-60 cells were treated with TSPf at increasing concentrations for 24 h, followed by immunoblotting against PARP and Caspase-3, two representative molecular markers of cell apoptosis. PARP was cleaved by TSPf in all AML cell lines examined (**Figure 4A**), and this cleavage was concentration- and time-dependent (**Figures 4B,C**). Consistent with the changes of PARP, Caspase-3 was activated by TSPf (**Figure 4D**). These results indicate that TSPf induce AML cell apoptosis.

### TSPf Induce Apoptotic Proteins and Downregulates Pro-survival Proteins

**FIGURE 5:**
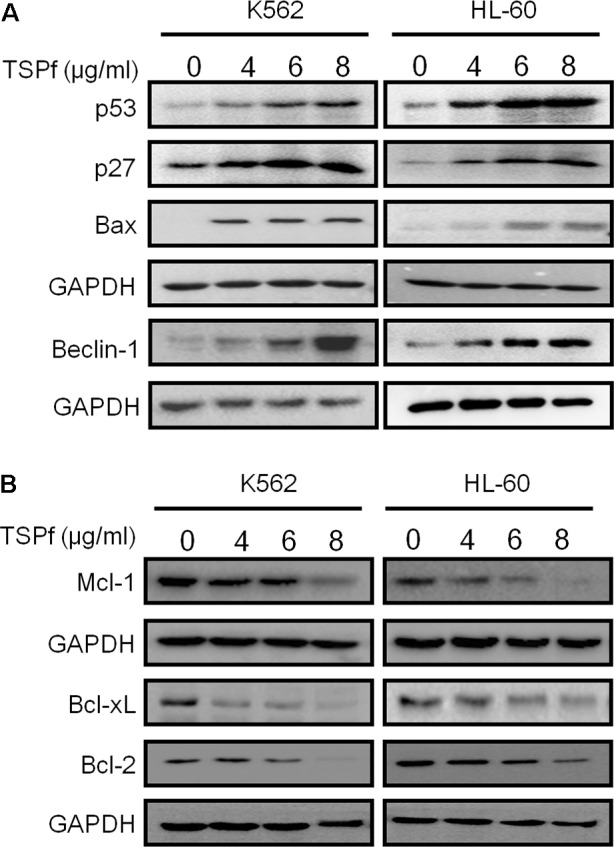
TSPf activates apoptotic signaling pathway and suppresses pro-survival genes. **(A)** Leukemia cell lines were treated with TSPf or DMSO for 24 h followed by lysate preparation and immunoblotting analysis against pro-apoptotic proteins as indicated. **(B)** The same cell lysates from A were applied for immunoblotting assay against pro-survival proteins as indicated. GAPDH was used as an internal loading control.

Apoptosis is a complicated cellular process including induction of pro-apoptotic genes and blockade of pro-survival genes. We subsequently evaluated the effects of TSPf on cellular survival and pro-death proteins in AML cells. As shown in **Figure 5A**, TSPf induced the expression of typical pro-death proteins, including p53, p27, Bax, and Beclin 1 (**Figure 5A**). In contrast, TSPf downregulated pro-survival proteins including Bcl-2, Bcl-xL, and Mcl-1 (**Figure 5B**). This finding further supported that TSPf induces AML cell apoptosis and prevented AML cells from survival.

### TSPf Suppresses the AKT/mTOR Signaling

**FIGURE 6:**
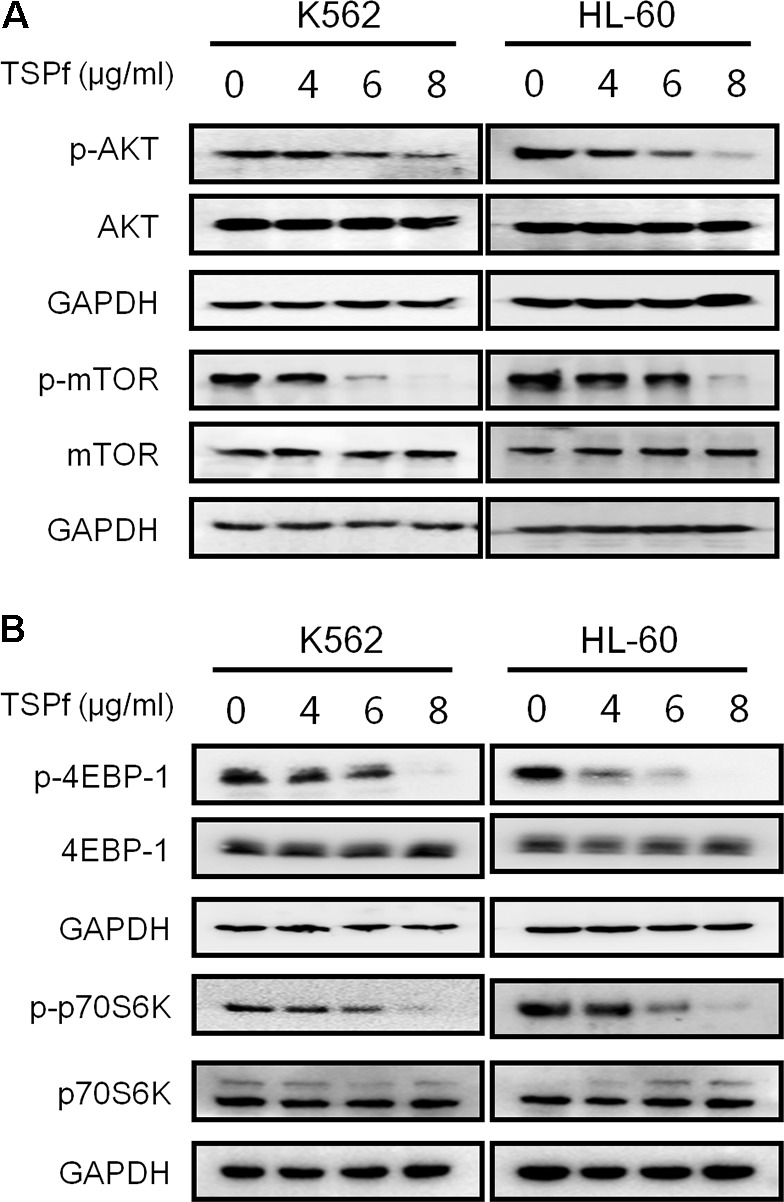
TSPf inhibits the AKT/mTOR signaling pathway. **(A)** K562 and HL-60 cells were treated with increasing concentrations of TSPf for 24 h. After incubation, cells were harvested and total proteins were isolated before being applied for immunoblotting assay against AKT and mTOR as indicated. **(B)** The same cell lysates from A were applied for immunoblotting assay against 4E-BP1 and p70S6K. GAPDH was used as an internal loading control.

The PI3K/AKT/mTOR signaling pathway is a central node of various signal transduction in AML initiation and progression (Fransecky et al., 2015). Over activation of AKT/mTOR correlates with poor prognosis of AML patients. The AKT/mTOR signaling pathway has been proposed as a potential therapeutic target for AML (Fransecky et al., 2015). Therefore, we tested whether TSPf suppressed the AKT/mTOR signaling transduction. As shown in **Figure 6A**, TSPf significantly inhibited the phosphorylation of both AKT and mTOR in both HL-60 and K562 cells, but had not changed their total protein expression levels. Consistent with the effects on AKT and mTOR, TSPf also suppressed the phosphorylation of 4EBP-1 and p70S6K, two key downstream signals of the AKT/mTOR signaling pathway (**Figure 6B**). Because phosphorylation levels are keys for the activation of these proteins, these results demonstrated that TSPf inhibited the AKT/mTOR signaling pathway.

### TSPf Downregulates RNF6 Expression

**FIGURE 7:**
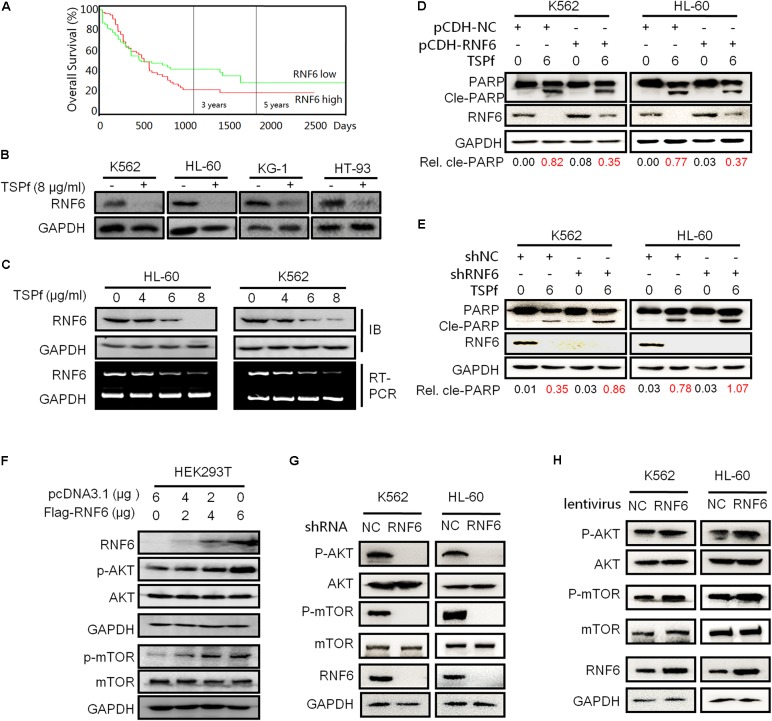
TSPf downregulates the RNF6/AKT/mTOR pathway. **(A)** The survival periods of leukemia patients were estimated using the Kaplan–Meier estimates as described in the Section “Materials and Methods.” All patients were classified into two groups based on the RNF6 expression level. Estimated survival percentage of each group of patients was calculated. **(B)** Leukemia cell lines were treated with 8 μg/ml TSPf or DMSO for 24 h followed by lysate preparation and immunoblotting (IB) analysis against RNF6 and GAPDH. **(C)** Leukemia cells (K562 and HL-60) were treated with increasing concentrations of TSPf for 24 h, followed by measurement of RNF6 protein by IB assay or mRNA expressions using RT-PCR. **(D)** K562 and HL-60 cells were infected with lentiviral RNF6, 96 h later, cells were treated with TSPf for 24 h. The cell lysates were then subjected to IB assay. The relative levels of cleaved PARP over total PARP were calculated by densitometry. **(E)** RNF6 was knocked down by shRNA from K562 and HL-60 cells, 96 h later, cells were treated with TSPf for 24 h. The cell lysates were then subjected to IB assay. The relative levels of cleaved PARP over total PARP were calculated by densitometry. **(F)** HEK293T cells were transfected with a RNF6 plasmid. Twenty-four hours later, cells were prepared for whole-cell lysates and subjected to IB against RNF6, AKT, p-AKT, mTOR, and p-mTOR. GAPDH was used as an internal loading control. **(G)** K562 and HL-60 cells were transfected with shRNF6 plasmids, 24 h later, cells were prepared for whole-cell lysates and subjected to immunoblotting against RNF6, AKT, p-AKT, mTOR, and p-mTOR. GAPDH was used as an internal loading control. **(H)** K562 and HL-60 cells were infected with lentiviral RNF6, 96 h later, cells were prepared for whole-cell lysates and subjected to immunoblotting against RNF6, AKT, p-AKT, mTOR, and p-mTOR. GAPDH was used as an internal loading control.

In our recent study, we found that RNF6 is overexpressed in AML but not in healthy adult bone marrow cells (Xu et al., 2016). Moreover, RNF6 plays a critical factor in AML cell proliferation, tumor progression, and chemoresistance (Xu et al., 2016). More importantly, RNF6 expression was found to be negatively associated with the overall survival of AML patients based on the AML data from the Cancer Genome Atlas (TCGA) (see footnote 2), the high expression of RNF6, the lower survival rate of AML patients as shown in **Figure 7A**. We thus wondered whether TSPf could downregulated the expression of RNF6 given its strong activity in inducing AML cell apoptosis. As shown in **Figure 7B**, RNF6 was decreased by TSPf in all cell lines. Moreover, TSPf downregulated RNF6 expression at both protein and RNA levels (**Figure 7C**). These results supported that TSPf inhibited RNF6 expression.

To find out whether RNF6 contributed to TSPf-induced leukemia cell apoptosis, both K562 and HL-60 cells were infected with lentiviral RNF6 or empty virus for 96 h, followed TSPf treatment. The IB evaluation on PARP cleavage indicated that overexpression of RNF6 partly ablated TSPf activity in inducing apoptosis (**Figure 7D**). Densitometric analysis showed that the ratios of cleaved PARP over total PARP were reduced owing to RNF6 expression in both cell lines (**Figure 7D**). In accordance with this finding, knockdown of RNF6 by shRNA enhanced leukemia apoptosis in both cell lines as shown in **Figure 7E**. Therefore, these data further supported that TSPf induced AML cell apoptosis by downregulating RNF6 expression.

### RNF6 Activates the AKT/mTOR Signaling Pathway

Because TSPf suppressed the AKT/mTOR signaling as shown in **Figure 6**, we tested whether there were any association between the AKT and the RNF6 pathways. HEK293T cells were overexpressed RNF6, followed by evaluation of the AKT/mTOR phosphorylation. As shown in **Figure 7F**, overexpression of RNF6 in HEK293T cells markedly stimulated the activation of both AKT and mTOR signals, however, the total protein levels of AKT and mTOR were not affected, which suggested that RNF6 probably triggered the phosphorylation of AKT and mTOR proteins. To test this hypothesis in leukemia cells, RNF6 was knocked down in both K562 and HL-60 cells, followed by the evaluation of the AKT signaling transduction. As shown in **Figure 7G**, when RNF6 was knocked down, AKT and mTOR phosphorylation was suppressed while their total protein expressions were not affected. Moreover, when RNF6 was overexpressed in these cells by lentivirus, AKT and mTOR were activated as seen their phosphorylation was induced (**Figure 7H**). Therefore, TSPf suppressed the activation of the AKT/mTOR signals by inhibiting RNF6 protein.

### TSPf Delays the Growth of AML Xenografts in a Nude Mouse Model

**FIGURE 8:**
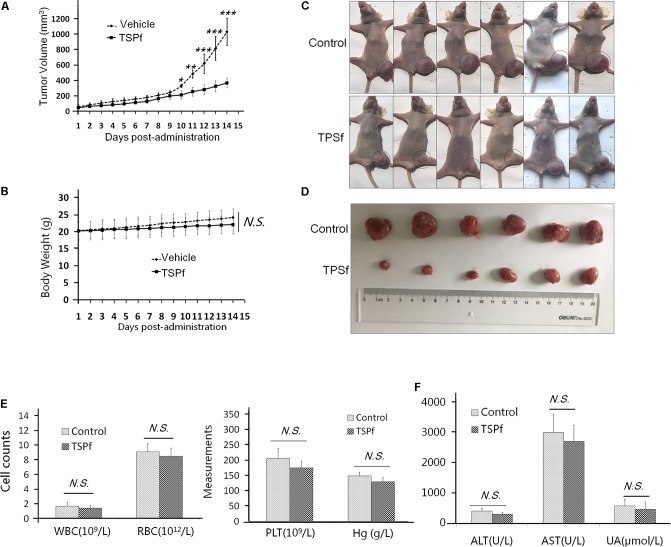
TSPf delays leukemia tumor xenograft growth in nude mice. **(A)** K562 cells were subcutaneously inoculated into the right flank of female nude mice to establish a leukemia xenograft model. When tumors were palpable, mice were divided randomly into two groups: one received TSPf orally (100 mg/kg body weight) on a daily basis for 14 days. During the experiments, tumor sizes were monitored every other day. ^∗^*p* < 0.05; ^∗∗^*p* < 0.01; ^∗∗∗^*p* < 0.001. **(B)** The body weight curve over the experiment. N.S.: not significant. **(C)** Mice tumors as shown in the dotted circles at the end of the experiments (two independent experiments were carried out, here showed one of them). **(D)** The tumor tissues dissected from mice at the end of the experiment. **(E)** Blood assays for white blood cells (WBC), red blood cell (RBC), platelets (PLT), and hemoglobin (Hg) at the end of the experiment. **(F)** Biochemical assays for alanine aminotransferase (ALT), aspartate aminotransferase (AST), and urine acid (UA) from blood at the end of the experiment.

To determine the anti-AML activity of TSPf *in vivo*, a subcutaneous AML xenograft model was established in nude mice with a human AML cell line K562. When tumors were palpable, the mice were randomly divided into two groups, one group was orally administered TSPf, the other was given vehicle as a control, for 14 days. As shown in **Figure 8A**, TSPf significantly slowed down the tumor growth in nude mice compared with the vehicle control over the TSPf treatment period. In the TSPf group, tumors were markedly decreased at the 11th day after TSPf was administrated. In the 14th day, the average tumor size in the treated group was less than 40% of the vehicle group. However, TSPf did not markedly affect mice body weights (**Figure 8B**). At the end of the experiment, all tumors from the TSPf-treated group were dramatically decreased as shown in **Figures 8C,D**. At the end of the experiment, the blood tests and biochemical assays were performed. It revealed that TSPf did not bring marked changes in blood count, classification, platelet number and function, hemoglobin measurement (**Figure 8E**). In the biochemical analyses on blood species from these mice, the results showed that TSPf did not alter the measurements in terms of alanine aminotransferase (ALT), aspartate aminotransferase (AST), blood urine nitrogen (UN) in TSPF-treated mice (**Figure 8F**). Therefore, TSPf was effective against AML without overt toxicity.

### TSPf Suppresses the RNF6/AKT/mTOR Signaling Axle *in Vivo*

**FIGURE 9:**
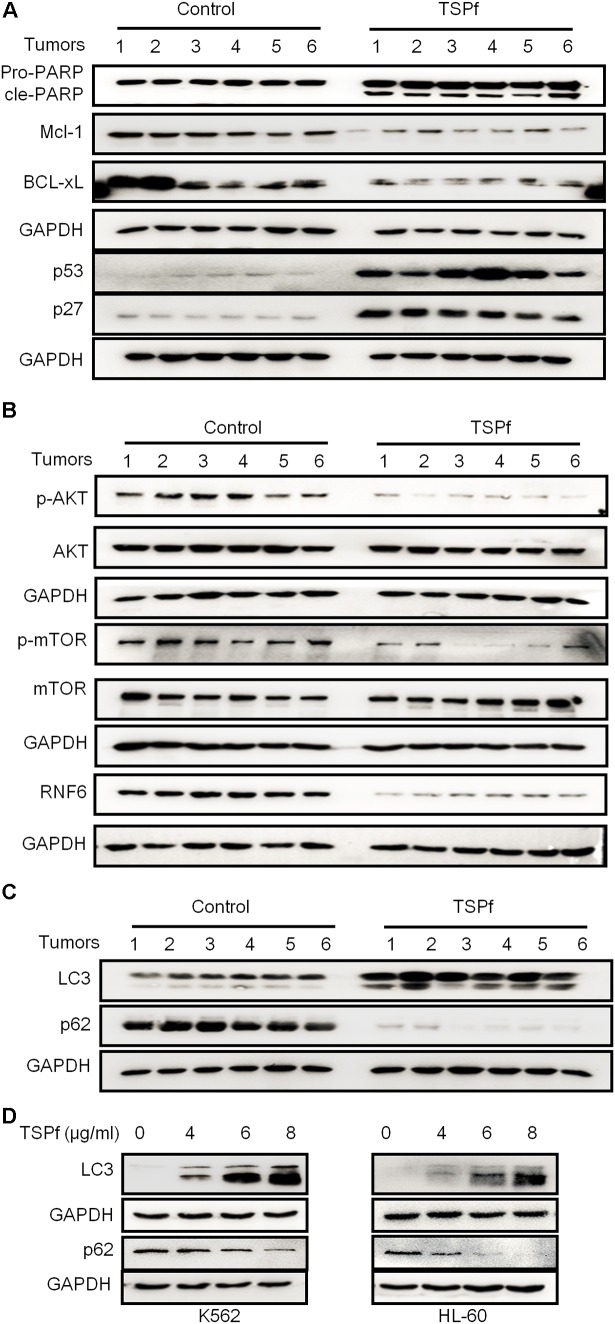
TSPf suppresses the RNF6/AKT/mTOR pathway in tumor tissues. At the end of the experiment, tumor tissues were dissected and frozen in liquid nitrogen immediately and stored at –80°C for further assays. Total proteins were extracted from the tumor tissues followed by immunoblotting assays against typical pro-survival and pro-apoptotic proteins **(A)**, the AKT and mTOR proteins and their phosphorylation levels **(B)**, the autophagic proteins LC3 and p62 **(C)**. **(D)** K562 and HL-60 cells were treated with TSPf at indicated concentration for 24 h, followed by immunoblotting for LC3 and p62.

The above findings have suggested that TSPf exerted anti-leukemia activity by targeting at the RNF6/AKT/mTOR signaling pathway based on the studies in cultured cells. To find out whether TSPf targets the same molecular events, we evaluated this pathway in tumor tissues from nude mice treated with TSPf. We measured the effects of TSPf in leukemia survival signals first. As shown in **Figure 9A**, TSPf induced the cleavage of PARP in tumors from TSPf-treated mice compared with the mock control group. Consistent with this change, pro-survival proteins such as Mcl-1 and BCL-xL were downregulated which pro-apoptotic proteins p53 and p27 were upregulated. These *in vivo* changes were similar to those found in the *in vitro* assay. Moreover, TSPf also downregulated the expression of RNF6 in the tumor tissues from mice treated with TSPf (**Figure 9B**), and the phosphorylation levels of both AKT and mTOR were suppressed by TSPf, but their total proteins were not altered (**Figure 9B**), which further demonstrated that TSPf displayed anti-AML leukemia activity by targeting at the RNF6/AKT/mTOR signaling pathway.

It is well known that mTOR is the switch of the autophagic pathway, which could promote cancer cell apoptosis, and mTOR activation was suppressed by TSPf, while Beclin 1, another critical molecule in autophagy, was induced, we wondered whether TSPf could trigger the autophagic process. To this end, we evaluated LC3 and p62, two key molecules in the autophagic flux, on tumor tissues treated by TSPf. As shown in **Figure 9C**, LC3-II was induced while p62 was decreased after TSPf treatment. To confirm this finding, AML cell lines K562 and HL-60 were treated with TSPf followed by IB assay to evaluate LC3 and p62. As shown in **Figure 9D**, TSPf treatment upregulated LC3-II and downregulated p62 in a concentration-dependent manner. Because mTOR is the very beginning signal in the autophagy process, LC3-II and Beclin 1 induction represents the intermediate events, while p62 degradation is a hallmark of the completion of autophagy, we could conclude that TSPf triggers the autophagic process.

## Discussion

*Paris forrestii* (Takht) H. Li is a flowering plant belonging to the Paris genus and it mainly grows in Tibet and Yunnan provinces in China. This plant has been long used for treating fever, headache, burns, wounds, bleeding by local inhabitants, but its function in the treatment of cancer is largely unknown. The present study demonstrated that TSPf from *Paris forrestii* (Takht.) H. Li displays potent anti-AML activity.

Saponins are a class of amphipathic glycosides structurally by having one or more hydrophilic glycoside moieties combined with a lipophilic triterpene derivative. Saponins have been widely found in various Chinese traditional herb medicines including soapberry, hippocastanaceae, ginseng, and Paris polyphyllin (Nagulapalli Venkata et al., 2017; Zhong et al., 2017). From the *n*-butanol extract, more than 10 major saponins were identified, including polyphyllin I, II, III, V, VII, PGRR, methyl-prosapogenin I and V, Pariposide A, β-ecdysone and others. Although most of these glycosides including polyphyllin I, II, H, and pariposide A are also found in *Paris polyphylla* var. *yunnanensis*, but their compositions are different (Wu et al., 2017). And polyphyllin III, methyl-prosapogenin A and E could be found in different species such as red ginseng, dioscorea and gleditsia japonica (Yoon and Kim, 2008; Lee et al., 2012). These findings indicated that *Paris forrestii* is different from other Paris species such as *Paris polyphylla* var. *yunnanensis*. This hypothesis was confirmed by the bioactivity of the total saponins from these two species of Paris in terms of AML cell proliferation. As shown in **Figure 2**, total saponins from *Paris forrestii* were more potent in suppressing AML cell proliferation compared with typical saponins and VP16. In addition, we found that TSPf displayed stronger anti-AML activity than individual saponins, which suggests that the individual saponins might synergize each other to induce AML cell apoptosis.

The underlying mechanisms of TSPf in inhibiting AML cell proliferation and inducing apoptosis was not reported. Our present study identified a novel mechanism that modulates the AKT/mTOR signaling pathway by RNF6. Targeting the PI3K/AKT/mTOR pathway is an emerging strategy for the treatment of hematological malignancies (Bertacchini et al., 2015). Recently two PI3K inhibitors idelalisib and copanlisib have been approved by US FDA for the treatment of various leukemia and lymphomas (Markham, 2017; Zhao et al., 2017). Although AKT inhibitors have not been approved for the treatment of leukemia, there are several undergoing clinical trials and great promising potentials have emerged (Lara et al., 2015). Saponins have been reported to inhibit the PI3K/AKT signaling pathway (Cui et al., 2017). In the present study, TSPf suppressed the phosphorylation and activation of the AKT signaling as well as its downstream signals but had no effects on their total expression, suggesting that TSPf inhibits the activation of the AKT signaling.

Interestingly, the present study found that TSPf suppresses the AKT/mTOR signaling transduction by inhibiting RNF6 transcription. RNF6 is a ring finger protein that has been proposed as a ubiquitin ligase by adding a ubiquitin moiety to the lysine residues of substrate proteins. For example, RNF6 mediates the polyubiquitination of androgen receptor and estrogen receptor thus increasing their activity and promotes cancer cell proliferation (Xu et al., 2009; Zeng et al., 2017). RNF6 was also found to be highly expressed in both primary AML bone marrow cells and AML cell lines (Xu et al., 2016). For example, RNF6 promotes K562 cell growth but when RNF6 is knocked down, AML cell proliferation is downregulated (Xu et al., 2016). More importantly, RNF6 increases the growth of AML xenograft derived from K562 cells, and clinically, RNF6 is negatively associated with the overall of AML patients. TSPf downregulated RNF6 expression at both mRNA and protein levels in association with AKT/mTOR inactivation. Overexpression of RNF6 upregulates the activation of the AKT/mTOR signaling, in contrast, interference with RNF6 using siRNA leads to downregulated AKT/mTOR signaling. This is the first report on RNF6 that activates the AKT/mTOR signaling, however, how the AKT/mTOR signaling pathway is activated by RNF6 deserves further investigation.

Taken together, the present study identified the active components in the root of *Paris forrestii* (Takht) H. Li, a traditional Chinese medicinal plant, by an HPLC-MS-NMR strategy. TSPf from the final *n*-butanol extract displays potent anti-AML activity. We also revealed a novel mechanism that TSPf targets at the RNF6/AKT/mTOR signaling axle. Given the insignificant toxicity and potent anti-AML activity *in vitro* and *in vivo*, TSPf could be further developed as a new therapy for AML patients.

## Author Contributions

SH and XM designed the study and analyzed the data. QL, YW, YZ, LG, YH, ZZ, QW, and BC conducted the experiments. QL wrote the manuscript. XM reviewed and revised the manuscript.

## Conflict of Interest Statement

The authors declare that the research was conducted in the absence of any commercial or financial relationships that could be construed as a potential conflict of interest.

## Footnotes

1 http://watson.compbio.iupui.edu/chirayu/proggene/database/index.php

2 https://tcga-data.nci.nih.gov/docs/publications/tcga/?

## Abbreviations

ALL: acute lymphoblastic leukemia
AML: acute myeloid leukemia
Bcl-2: B-cell lymphoma-2
DMSO: dimethyl sulfoxide
DNMT1: DNA (cytosine-5)-methyltransferase 1
EZH2: enhancer of zeste homolog 2
HPLC: high performance liquid chromatography
Mcl-1: myeloid cell leukemia-1
MTT: 3-(4,5-dimethylthiazol-2-yl)-2,5-diphenyltetrazolium bromide
NMR: nuclear magnetic resonance
PI: propidium iodide
RNF6: ring finger protein 6.
